# Variability in Behavioral Phenotypes after Forced Swimming-Induced Stress in Rats Is Associated with Expression of the Glucocorticoid Receptor, Nurr1, and IL-1β in the Hippocampus

**DOI:** 10.3390/ijms222312700

**Published:** 2021-11-24

**Authors:** Elizabeth Ruiz-Sánchez, Arely M. López-Ramírez, Ángel Ruiz-Chow, Minerva Calvillo, Aldo A. Reséndiz-Albor, Brenda Anguiano, Patricia Rojas

**Affiliations:** 1 Laboratory of Neurotoxicology, National Institute of Neurology and Neurosurgery Manuel Velasco Suárez, SS, Av. Insurgentes Sur No. 3877, Mexico City C.P. 14269, Mexico; elizabeth.ruiz@innn.edu.mx (E.R.-S.); arelymlopezr@gmail.com (A.M.L.-R.); 2 Neuropsychiatry Unit, National Institute of Neurology and Neurosurgery Manuel Velasco Suárez, SS, Av. Insurgentes Sur No. 3877, Mexico City C.P. 14269, Mexico; angel.ruiz@innn.edu.mx; 3 Experimental Laboratory of Neurodegenerative Diseases, National Institute of Neurology and Neurosurgery Manuel Velasco Suárez, SS, Av. Insurgentes Sur No. 3877, Mexico City C.P. 14269, Mexico; calvel_mine@hotmail.com; 4 Mucosal Immunity Laboratory, Research and Graduate Section, Instituto Politécnico Nacional, Escuela Superior de Medicina, Plan de San Luis Esq. Salvador Díaz Mirón s/n, Mexico City C.P. 11340, Mexico; alrealdo@yahoo.com.mx; 5 Instituto de Neurobiología, Universidad Nacional Autónoma de México (UNAM), Campus Juriquilla, Boulevard Juriquilla 3001, Querétaro C.P. 76230, Mexico; anguianoo@unam.mx

**Keywords:** behavioral phenotype, individual differences, glucocorticoid receptor, Nurr1, IL-1β, vulnerable phenotype, resilient phenotype, stress resilience

## Abstract

Individual differences in coping with stress may determine either a vulnerable or resilient phenotype. Therefore, it is important to better understand the biology underlying the behavioral phenotype. We assessed whether individual behavioral phenotype to acute stress is related with the hippocampal expression of glucocorticoid receptor (GR), Nurr1, interleukin-1 beta (IL-1β) or brain-derived neurotrophic factor (BDNF). Wistar male rats were exposed to forced swimming for 15 min and sacrificed at different times. Behavioral response was analyzed, and it was compared with the gene and protein expression of GR, Nurr1, IL-1β and BDNF in the hippocampus for each time point. Behavioral phenotyping showed a group with high immobility (vulnerable) while another had low immobility (resilient). No significant differences were found in the *Nurr1*, *IL-1β* and *BDNF* mRNA levels between resilient and vulnerable rats at different recovery times except for *Nr3c1* (gene for GR). However, exposure to stress caused significantly higher levels of GR, Nurr1 and IL-1β proteins of vulnerable compared to resilient rats. This variability of behavioral phenotypes is associated with a differential molecular response to stress that involves GR, Nurr1, and IL-1β as mediators in coping with stress. This contributes to identifying biomarkers of susceptibility to stress.

## 1. Introduction

Stress is a potential risk factor related to the development of psychiatric disorders such as depression and post-traumatic stress disorder that alter the quality of life [1]. Nonetheless, it is known that the behavioral response to stress is influenced by diverse factors such as the environmental context, the intensity, frequency, type and duration of the stressful stimulus [2]. Psychological stress due to a traumatic event has long-lasting effects on behavior that leads to the development of adaptive or coping mechanisms in individuals, which are associated with memories linked to strong emotions [3]. Although the majority of the population can adapt successfully, between 7% to 15% of people can develop a stress-related disorder [4]. Nowadays, it is accepted that an individual’s genetic predisposition may contribute either resilience or vulnerability to developing stress-induced mental disorders [2,5].

It is well-known that the hypothalamic-pituitary-adrenal (HPA) axis is activated in response to stressful stimuli, which results in the secretion of glucocorticoid hormones [6]. Glucocorticoids action in the brain is performed through binding and activation of two types of receptors, the glucocorticoid receptor (GR) and mineralocorticoid (MR) receptor [6]. In particular, it has been widely reported that the glucocorticoid-GR system plays a pivotal role in the stress response [6] through genomic processes that depend on GR-mediated transcription as well as de novo protein synthesis. Therefore, depending on the transcriptional activity characteristic of this system in response to stressful stimuli, it may induce maladaptive brain changes associated with vulnerability, or it may induce cerebral programming that allows adaptive coping (resilience) [2,7,8,9]. GRs are ubiquitously expressed with high density in the hippocampus, a brain region relevant to the stress response. Once activated, these receptors move into the nucleus to act on target genes to inhibit (sometimes) or facilitate transcription [10]. However, the non-genomic action of glucocorticoids through membrane-bound receptors have also been reported [10].

Also, it has been suggested that Nurr1, a transcription factor belonging to orphan nuclear receptors may be an important mediator in the stress response because it is sensitive to glucocorticoids and plays a key role in adaptive responses to stress. It is related to its transcriptional activity that regulates the neuroendocrine response of the HPA axis [11,12,13]. In particular, a synergistic interaction between Nurr1 and GR has been reported in the hippocampus resulting in the formation of the Nurr1-GR heterodimer [14].

In behavioral studies, we have reported that *Nurr1* heterozygous (+/−) mice showed hyperactivity and vulnerability in the stress response induced by the forced swimming test [15,16]. An increase of *Nurr1* mRNA expression paralleled by an enhancement of the protein was also demonstrated in the hippocampus of mice, after the forced swimming test [17]. Furthermore, chemical stress can increase *Nurr1* mRNA but decrease protein synthesis in the hippocampus [18]. Other authors have shown an increase of *Nurr1* in the hypothalamus and the pituitary gland of mice after restraint stress [13,19].

Stress-induced neuroinflammation has been related with psychiatric disorders [20,21,22]. Recent studies have shown that proinflammatory cytokines, such as interleukin-1 beta (IL-1β), induced by stress, initiate the neuroinflammatory processes producing the physiological and behavioral responses associated with mental disorders [20,21,22]. Indeed, it has been reported that the administration of IL-1β induces depressive-like behavior in rodents [23,24]. Evidence has also shown a relation between Nurr1 and proinflammatory stimuli [25]. It is known that Nurr1 senses and increases rapidly in response to such stimuli [25,26]. Furthermore, it has been showed that Nurr1 regulates the expression of corticotropin-releasing factor and proopiomelanocortin (mediators of the HPA axis) in response to proinflammatory cytokines [27,28]. In addition, Nurr1-mediated inflammatory signaling has been shown to repress IL-1β expression [29].

Another important factor that has been implicated in stress-related disorders is the brain-derived neurotrophic factor (BDNF) [30]. It is known that the decrease in this neurotrophin induces vulnerability whereas its adequate level promotes adaptive plasticity mechanisms, in response to stress [30,31]. Moreover, BDNF is a Nurr1 target gene with a direct effect of Nurr1 on the transcriptional activity of different BDNF promoter regions [32].

It is important to mention that forced swimming-induced stress is a stressful psychological event that gives us the opportunity to study the role of glucocorticoids and their receptors in the brain related to the behavioral immobility response, as previously reported [33]. It is also used to evaluate performance on immobility scores, as a criterion to test animals’ vulnerability, since stress response differs between individuals [34].

Although knowledge of the expression of genes that participate in the development of stress-induced disorders has advanced, this has not been studied widely in relation to individual differences. Here we tested the hypothesis whether the exposure to acute stress, induced by forced swimming, shows different behavioral phenotypes, and whether it is related with a specific expression profile of molecules associated to stress response such as GR, Nurr1, IL-1β or BDNF in the hippocampus as mediators of a specific behavioral phenotype, vulnerable or resilient.

## 2. Results

### 2.1. Behavioral Response

The rats were classified as vulnerable (high immobility) or resilient (low immobility) for each of the recovery times (0, 30 min, 3 h, 24 h and Test + 30 min), according to the immobility time during the first 15 min of forced swimming as described in the method section.

**Figure 1:**
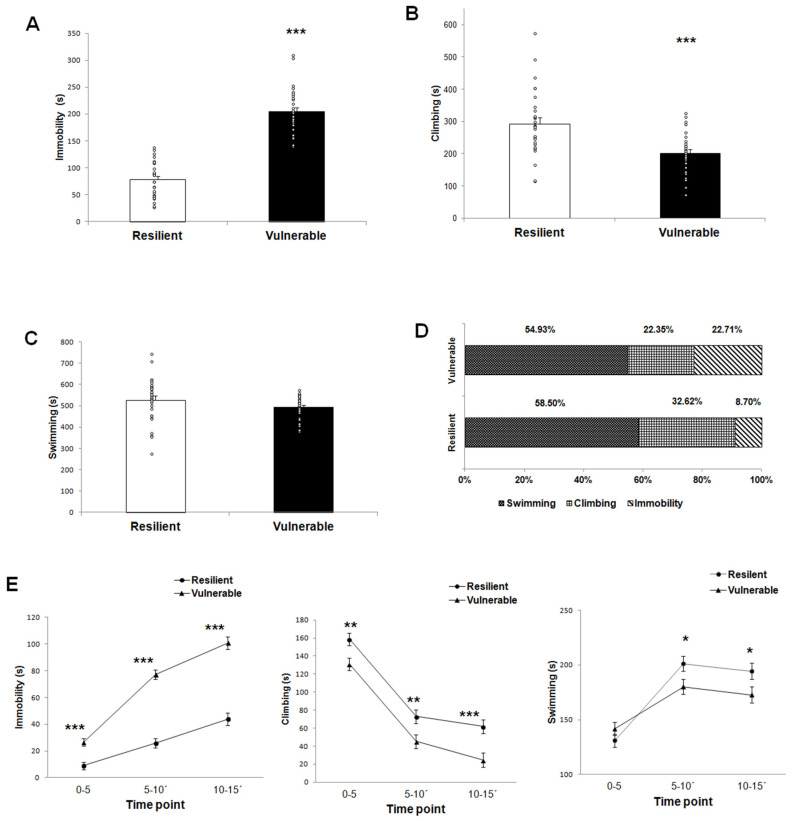
The variability in behavioral phenotypes (vulnerable vs. resilient) to forced swimming-induced acute stress is associated with different behavioral response. (**A**) The vulnerable group had a higher total time of immobility than the resilient group. (**B**) The vulnerable group showed a lower total time of climbing time than the resilient group. (**C**) There was no significant difference between the vulnerable and resilient groups in total time of swimming. (**D**) Percentage of the three behaviors analyzed for the two behavioral phenotypes (vulnerable vs. resilient). (**E**) Behavioral analysis throughout the 15 min of forced swimming-induced acute stress. The vulnerable group had a higher immobility time than the resilient group, during the first 5 min, 10 min, and 15 min of forced swimming. The vulnerable group showed lower climbing time than the resilient group for the different times analyzed. The vulnerable group showed lower swimming time than the resilient group during the first 10 min and 15 min of forced swimming. Data are expressed as the mean ± SEM. Number of rats per group: Vulnerable n = 29; resilient n = 29. Data for A, B and C were analyzed using unpaired *t*-tests. Data E were analyzed by two-way repeated-measures ANOVA followed by Bonferroni *post hoc* tests ᵒ individual values. * *p* < 0.05, ** *p* < 0.01 and *** *p* < 0.001 indicate significant differences between the resilient and vulnerable groups.

The total time for immobility, climbing, and swimming for the 15 min of forced swimming was compared between behavioral phenotypes. We found a significant difference between resilient and vulnerable groups in the total time of immobility (t (56) = 12.62, *p* = 0.000; Figure 1A) and climbing (t (56) = 4.13, *p* = 0.000) (Figure 1B). There was no significant difference in the total time of swimming between the two behavioral phenotypes (t (56) = 1.495, *p* = 0.142) (Figure 1C).

The percentage of the three behaviors analyzed for the two behavioral phenotypes showed that vulnerable rats spent more time in passive behavior (immobility) than resilient rats (22.71% in vulnerable vs. 8.70% resilient) but less time in active behaviors (swimming + climbing, 77.28%) when compared to the resilient group (91.12%) (Figure 1D). In addition, the total time for immobility, climbing, and swimming for the 15 min of forced swimming was compared between behavioral phenotypes.

A two-way repeated measures ANOVA showed a main effect on the individual behavioral phenotype (vulnerable vs. resilient) and time (0–5 min, 5–10 min, and 10–15 min) for passive and active behaviors identified in 15 min of forced swimming. The statistical values for immobility (passive behavior) included the main effect on the phenotype (F(1,56) = 159.4, *p* = 0.000) and time (F(1.55,87.091) = 117.99, *p* = 0.000); an interaction between phenotype × time (F = (1.55,87.091) = 17.48, *p* = 0.000), as well as significant differences between resilient and vulnerable groups at 0–5 min (t^2^ = 19.23, *p* = 0.000), 5–10 min (t^2^ = 105.39, *p* = 0.000) and 10–15 min (t^2^ = 74.53, *p* = 0.000) in *post hoc* comparisons with Bonferroni corrections. The vulnerable group had a significantly higher immobility time compared to the resilient group throughout the 15 min test (Figure 1E).

For climbing (active behavior), repeated measures ANOVA exhibited a significant main effect on behavioral phenotype (F (1,56) = 17.039, *p* = 0.000) and time (F (2,112) = 143.598, *p* = 0.000), while no interactions were found for phenotype x time (F (2,112) = 0.342, *p* = 0.711). The *post hoc* analysis revealed that resilient animals spent more time climbing than the vulnerable group during 15 min of forced swimming (0–5 min, t^2^ = 8.14, *p* = 0.006; 5–10, t^2^ = 6.52, *p* = 0.013; 10-15, t^2^ = 11.26, *p* = 0.001) (Figure 1E).

Regarding the swimming (active behavior), a significant main effect was found in the variable of time (F (1.851,103.676) = 57.045, *p* = 0.000). Interaction between behavioral phenotype x time was found for swimming behavior (F (1.851,103.676) = 5.627, *p* = 0.006), but no main effect on behavioral phenotype (F (1,56) = 2.235, *p* = 0.141). *Post hoc* analysis revealed significant differences among the resilient and vulnerable group at two time points: 5–10 min (t^2^ = 4.71, *p* = 0.034) and 10–15 min (t^2^ = 4.24, *p* = 0.044). The resilient group had a significantly higher swimming time than the vulnerable group at two time points analyzed (Figure 1E).

**Figure 2:**
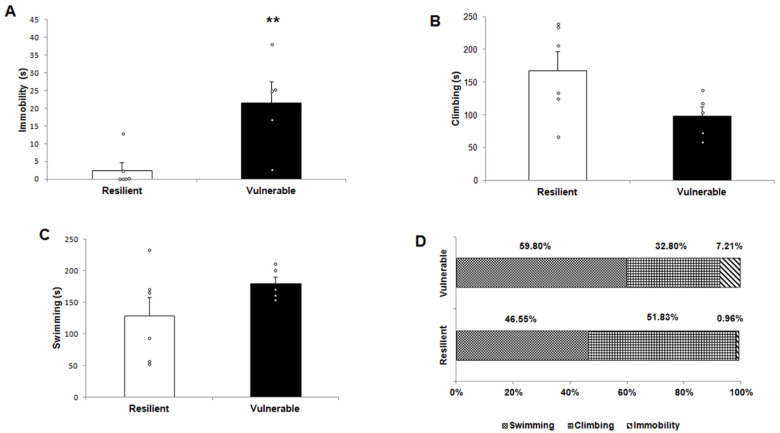
A second exposure of forced swimming at 24 h after the first exposure of 15 min also showed that the vulnerable group spent more time in passive behavior and lower in active behaviors than resilient rats. (**A**) The vulnerable group had a higher immobility time than the resilient group. (**B**) The vulnerable group showed a trend of lower climbing time than the resilient group. (**C**) There were no significant differences between the vulnerable and resilient groups in swimming. (**D**) Percentage of the three behaviors analyzed for the two behavioral phenotypes (vulnerable vs. resilient). Data are expressed as the mean ± SEM. Number of rats per group: vulnerable n = 5; resilient n = 6. Data were analyzed using unpaired *t*-tests. ᵒ individual values. ** *p* < 0.01 indicates significant differences between the resilient and vulnerable groups.

The group with a second test of forced swimming at 24 h after the first exposure of 15 min (Test + 30 min) was also recorded, Figure 2. The behavioral evaluation in this second session also showed a significant differential behavioral response between vulnerable and resilient phenotypes for the immobility parameter (t _(11)_ = −3.329, *p* = 0.009) (Figure 2A) and a trend for climbing (t _(11)_ = 2.193, *p* = 0.063) (Figure 2B). The vulnerable group had a significantly higher immobility time with respect to the resilient group throughout the 5 min test. There was no significant difference in swimming time between the two behavioral phenotypes (t _(11)_ = −1.614, *p* = 0.155) (Figure 2C). In this sense, the vulnerable rats spent more time in the passive behavior (immobility: 7.21% in vulnerable vs. 0.96% resilient) and less time in active behavior (swimming + climbing, 92.6%) than resilient rats (98.38%) in the percentage of the three behaviors recorded (Figure 2D).

Taken together, our findings support the behavioral classification according to animals’ stress response.

### 2.2. Plasma Corticosterone Concentration

**Figure 3:**
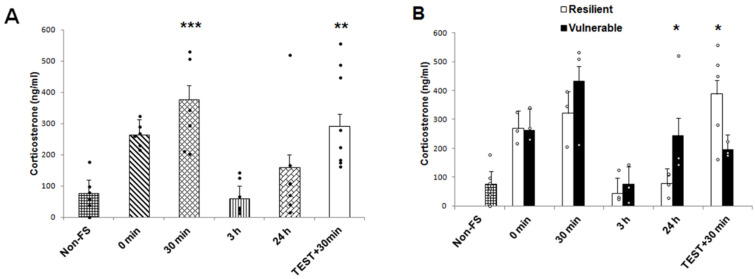
Plasma corticosterone concentrations after different recovery times following acute stress induced by forced swimming test. (**A**) Comparison between the different recovery times for the FS group; 6–8 rats per group, • individual values, ** *p* < 0.01 and *** *p* < 0.001 compared to Non-FS animals. (**B**) Responses differed between the vulnerable and resilient rats at different recovery times; 3–5 rats per group, ᵒ individual values. * *p* < 0.05 indicates significant differences between the resilient and vulnerable groups. Differences were analyzed with a two-way ANOVA followed by Bonferroni *post hoc* tests. Data are expressed as the mean ± SEM. FS, forced swimming.

Plasma corticosterone concentrations were quantified to analyze the neuroendocrine response after acute stress induced by forced swimming, and to analyze whether this response differed between the vulnerable and resilient rats. A two-way ANOVA revealed significant effects on recovery times (0 min, 30 min, 3 h, 24 h, Test + 30 min) (F (4,28) = 8.344, *p* = 0.000). However, no significant effect for the individual behavioral phenotype (resilient and vulnerable) (F (1,28) =0.316, *p* = 0.578) was reported but an interaction for phenotype × recovery time was found (F (4,28) = 2.933, *p* = 0.038). *Post hoc* multi-comparison tests showed that the plasma corticosterone concentration was significantly increased at 30 min (t^2^ = 22.38, *p* = 0.002) as well as in the “Test + 30 min” group (t^2^ = 13.54, *p* = 0.015) in the rats exposed to acute stress by forced swimming, as compared to non-stressed rats. At 0 min, 3 h and 24 h of recovery, plasma corticosterone concentrations were at basal levels (Figure 3A). We identified a significant difference between resilient and vulnerable groups at 24h and “Test + 30 min” after using Bonferroni *post hoc* test (Figure 3B). Corticosterone levels increased in the vulnerable group when compared to the resilient group at 24 h (t^2^ = 4.19, *p* = 0.05). In contrast, corticosterone levels increased in the resilient compared to the vulnerable animals in “Test + 30 min” recovery time group (t^2^ = 6.12, *p* = 0.02). There were no significant differences among phenotypes concerning other recovery times (0 min, t^2^ = 0.04, *p* = 0.948; 30 min, t^2^ = 1.43, *p* = 0.242; 3 h, t^2^ = 0.14, *p* = 0.710).

### 2.3. mRNA and Protein Expression

A two-way ANOVA analysis was conducted to assess the effects of recovery time (0, 30 min, 3 h, 24 h, Test + 30 min) and behavioral phenotype (resilient and vulnerable), as well as the interaction of these two factors on expression of GR, Nurr1, IL-1β, and BDNF (mRNA and protein) in response to acute stress induced by forced swimming.

**Figure 4:**
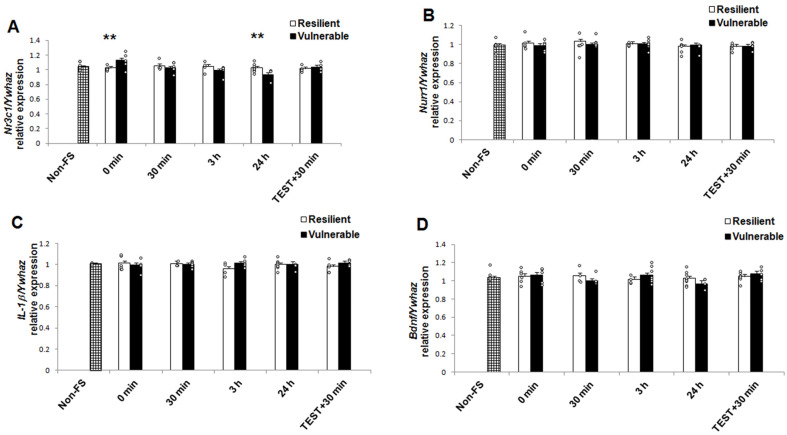
Vulnerability to forced swimming-induced acute stress is associated with higher *Nr3c1* mRNA levels than resilience after 24 h of recovery from acute stress (panel 4**A**) in hippocampus. There were no differences in the levels of *Nurr1, IL-1β, and BDNF* mRNA between the two groups at any of the times analyzed (panel 4**B**–**D**). Data are expressed as the mean ± SEM. 4–9 rats per group. Differences were analyzed with a two-way ANOVA followed by Bonferroni *post hoc* tests. ** *p* < 0.01 compared to the respective resilient group. *Nr3c1*, the gene that encodes for glucocorticoid receptor; *BDNF*, brain-derived neurotrophic factor; *IL-1β*, interleukin-1 beta; ᵒ individual values.

The results of two-way ANOVA analysis for mRNA and protein are presented in Table 1. Both behavioral phenotype and recovery time as well as the interaction of these factors had no significant effect on the *IL-1β, Nurr1,* and *BDNF* mRNA levels. There was no statistically significant difference in the *IL-1β, Nurr1,* and *BDNF* mRNA levels in the hippocampus between resilient and vulnerable groups, at any of the recovery times (Figure 4B–D). However, in the mRNA expression of *Nr3c1* (the gene that encodes for GR), a two-way ANOVA revealed significant main effects of recovery time, but no significant main effect relating to individual behavioral phenotype. In addition, an interaction for individual behavioral phenotype x recovery time was reported (Table 1). We identified a significant difference between resilient and vulnerable animals at 0 min and 24h (Bonferroni *post hoc* test; Figure 4A). *Nr3c1* mRNA levels were lower in resilient compared to vulnerable animals at 0 min (t^2^ = 8.83, *p* = 0.004). In contrast, *Nr3c1* mRNA levels increased in resilient compared to vulnerable animals at 24 h (t^2^ = 6.06, *p* = 0.018). There was no significant difference among phenotypes concerning other recovery times (30 min, t^2^ = 0.65, *p* = 0.428; 3 h, t^2^ = 1.78, *p* = 0.193; Test + 30 min, t^2^ = 0.37, *p* = 0.546).

**Figure 5:**
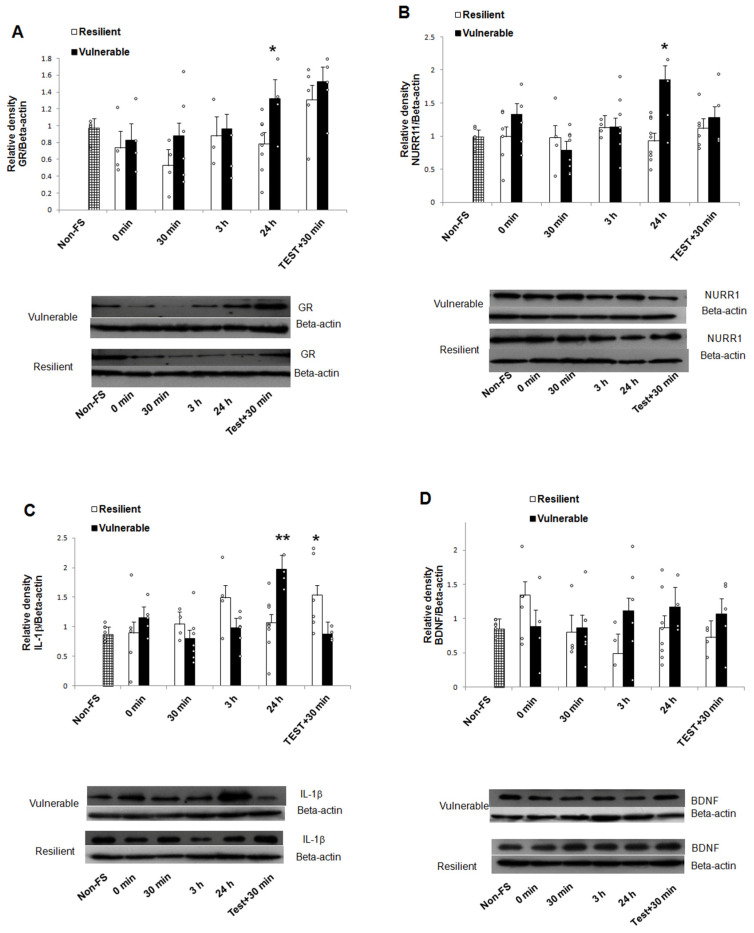
Vulnerability to forced swimming-induced acute stress is related with the increased expression of hippocampal GR, Nurr1, and IL-1β proteins. Vulnerable rats had higher levels of GR, Nurr1 and IL-1β GR in the hippocampus compared to resilient rats, at 24 h of recovery from the first exposure to acute stress (panel **A**–**C**). There were no differences in the levels of hippocampal BDNF proteins between the two groups at any of the times analyzed (panel **D**). Representative western blots of the expression of GR (95 KDa), Nurr1 (66 KDa), IL-1β (30 KDa), BDNF (15 KDa), and the Beta-actin (43 KDa) control are shown in the corresponding graphs. Data are expressed as the mean ± SEM. 3-8 rats per group. Differences were analyzed with a two-way ANOVA followed by Bonferroni *post hoc* tests. * *p* < 0.05 and ** *p* < 0.01 compared to the respective resilient group. BDNF, brain-derived neurotrophic factor; GR, glucocorticoid receptor; IL-1β, interleukin-1 beta; ᵒ individual values; * statistical significance at 0.05; ** statistical significance at 0.01.

The analysis of protein expression (Table 1) using two-way ANOVA identified: (a) in GR, a significant main effect of recovery time and behavioral phenotype; (b) in Nurr1, a significant main effect of behavioral phenotype as well as interaction of behavioral phenotype x recovery time; (c) in IL-1β, significant main effect of recovery time and interaction of behavioral phenotype x recovery time. The comparison of protein expression (Bonferroni test) showed that exposure to forced swimming caused significantly higher levels of GR, Nurr1, and IL-1β in the hippocampus of vulnerable rats compared to the resilient rats after 24 h of recovery (GR, t^2^ = 4.03, *p* = 0.05; Nurr1, t^2^ = 13.77, *p* = 0.000; IL-1β, t^2^ = 10.61, *p* = 0.002; Figure 5A–C). No differences were found at any of the other recovery times for GR and Nurr1 (GR: 0 min, t^2^ = 0.11, *p* = 0.742; 30 min, t^2^ = 2.06, *p* = 0.152; 3 h, t^2^ = 0.08, *p* = 0.759; Test + 30 min, t^2^ = 0.73, *p* = 0.397; Nurr1: 0 min, t^2^ = 2.13, *p* = 0.149; 30 min, t^2^ = 0.65, *p* = 0.423; 3 h, t^2^ = 0.05, *p* = 0.940; Test + 30 min, t^2^ = 0.53, *p* = 0.469). However, IL-1β protein levels increased in resilient compared to vulnerable animals in the “Test + 30 min” recovery time group (t^2^ = 5.96, *p* = 0.018). The levels of hippocampal BDNF were not significantly different between the vulnerable and the resilient rats, at any of the times studied (0 min, t^2^ = 2.07, *p* = 0.156; 30 min, t^2^ = 0.04, *p* = 0.840; 3 h, t^2^ = 3.28, *p* = 0.076; 24 h, t^2^ = 0.82, *p* = 0.369; Test + 30 min, t^2^ = 1.07, *p* = 0.305) (Figure 5D).

## 3. Discussion

In the current study we identified two different behavioral phenotypes in response to a single forced swim session in animals exposed for first time to this novel challenge. One group of rats had a high immobility time and thus was classified as vulnerable, while another group had a low immobility time and thus was called resilient. The diverse behavioral responses due to individual differences in coping with stress is consistent with other studies [34,35,36,37,38]. In our study, rats were exposed to a single forced swim test of 15 min to identify individual behavioral vulnerability to develop behavioral despair according to immobility scores. This reflects the ability of rodents to cope with a novel environment that can lead to the modulation of behavior in subsequent exposures.

Therefore, we also analyzed the immobility scores in a second 5-min forced swimming test 24 h after the first session. The percentage of passive behavior (immobility) was higher during the first exposure to the forced swim, both in vulnerable and resilient groups when compared to the second stress session. Nonetheless, the percentage of active behavior (swimming plus climbing) was lower in the first exposure to stress both in vulnerable and resilient animals as compared to the second exposure to the forced swim. This suggests the contribution of consolidated memory processes in the second exposure and a very important reason for classifying the individual behavioral phenotypes using immobility scores from the first 15-min forced swim session, because the rodents were exposed to a novel environment to evaluate coping strategies [34]. Nevertheless, we should realize that the different behavioral phenotype of rodents results, in part, from association of genetic and environmental factors which must be considered.

The study of individual differences is crucial to understanding why some individuals are resilient and others vulnerable to stress stimuli. Furthermore, the first forced swimming session provides the opportunity to elucidate the molecular mechanisms underlying the behavioral pattern induced by stress, making it possible to widen an understanding of the vulnerability to the development of stress-related disorders.

Acute stress responses, such as to the forced swim test, are mainly regulated by the HPA axis [39], and its activation releases glucocorticoids, a crucial part of the stress-response to face the metabolic challenge. Glucocorticoids play an important role in stress-coping as well as behavioral adaptation [31]. Thus, corticosterone levels in the current study showed that this hormone increased in the vulnerable group compared to the resilient group 24 h after the first exposure to forced swimming. On the contrary, corticosterone levels were enhanced in the resilient vs. vulnerable group after the second exposure to forced swim test. It is a very interesting finding that could be related to the circulating levels of corticosterone in the blood that remained longer in the vulnerable group, with a different response to a second challenge of stress, compared to the resilient group, so that the latter group could cope better with the stressful situation as seen in the reduction of immobility time. Also, this might be related to the GR response by genomic (transcriptional) and non-genomic mechanisms (intracellular signaling, epigenetics) that can alter the immediate response or the reactivity to future stress by mechanisms such as epigenetics [10].

The hippocampus plays a relevant role in the detection of novel stimuli, as well as a guide in behavioral responses based on familiarity [40]. This important brain region is strongly associated with the development of stress-related disorders [41]. Therefore, research into the molecular changes in hippocampus associated with a specific behavioral phenotype is a priority. In our study, we observed that the expression of *Nr3c1* mRNA (gene that encodes for GR), in the first exposure to stress, increased significantly at 0′ min in the vulnerable group compared to the resilient group. However, at 24 h after the first exposure to forced swimming there was a decrease in the vulnerable group compared to the resilient group. In regard to GR protein expression, we found an increase at 24 h after the first exposure to the stressor in the vulnerable group versus the resilient group.

Our results show differences between two times analyzed for both *Nr3c1* mRNA and GR protein. This difference between the resilient and vulnerable groups may also be related to genomic responses of GR in the hippocampus due to a corticosterone threshold and other mechanisms that are independent of the circulating levels of this hormone, as previously suggested [42]. It is important to emphasize that GR can modify neuronal function also by non-genomic mechanisms. It is also relevant to mention that these mechanisms are immediate, thus the changes in *Nr3c1* mRNA and GR protein in the first minutes after the first exposure to stress can be related, for example, to epigenetic modifications of cytosine bases in DNA, post-translational modifications of histones that have been previously reported for GR [10,43] which are mechanisms that are capable of mediating molecular changes and duration of the stress response.

In this sense, the decrease of *Nr3c1* mRNA levels in resilient rats was not accompanied by a change in GR protein at time 0 after the first exposure of forced swimming. This could be related to DNA methylation of the *Nr3c1* gene as well as an increase of miR-124a expression, as previously reported for this behavioral test [44,45], that could be reflected only at the mRNA level. Furthermore, the decrease in *Nr3c1* mRNA expression levels in vulnerable rats at the same time as an increase in corticosterone at 24 h after the first exposure to the stress stimulus, may be related to an inability to cope with stress of vulnerable rats as reported previously [11].

In addition, it is known that the transcription factor Nurr1 is sensitive to glucocorticoids and plays a key role in adaptive responses to stress because it is capable of directly regulating the transcription of target genes in the HPA axis [46]. However, Nurr1 is also expressed in brain regions such as substantia nigra, nucleus accumbens, the prefrontal cortex and the hippocampus [46,47] where it also exerts actions.

In the present study, we found no differences in *Nurr1* mRNA at the different times studied. Likewise, we found that after the first exposure to forced swimming, there was an increase in the Nurr1 protein at 24 h in the vulnerable group, as occurred in the GR protein. These results reflect the association that exists between these two nuclear receptors, Nurr1 and GR in the hippocampus, with a synergistic interaction in the Nurr1 response element, thus forming a Nurr1-GR heterodimer [14]. Thus, regulation of the transcriptional activity of Nurr1 due to glucocorticoids is through physical and functional interaction with GR. However, this association may be influenced by the cellular environment in which co-factors that modulate the transcriptional response may be participating.

It is important to mention that we did not find an increase in *Nurr1* mRNA at the different times analyzed after the first exposure of forced swimming for 15 min, although it has been previously reported that the expression of *Nurr1* mRNA increases at 30 min and at 3 h after a second exposure to forced swimming at 24 h after the first exposure [17]. However, we found no difference in *Nurr1* mRNA after the second exposure to forced swimming. This could be related to the fact that the behavioral test was performed in different species of rodents, because in the present study we used rats and in the mentioned study mice were used. Also, the *Nurr1* mRNA was analyzed in the total hippocampus that differs to the analysis for each subregion of the hippocampus as previously reported [17]. This may have contributed to the underestimation of probable undetected differences in the total hippocampus analysis.

In regards to the difference in the expression of Nurr1 protein found between vulnerable and resilient groups, this may be associated to Nurr1 total protein analyzed, which did not distinguish between the cytoplasmic and nuclear protein in order to know whether the active form associated to the Nurr1-GR heterodimer is participating in the expression of the different behavioral phenotypes.

Additionally, clinical and basic research studies suggest that stress-induced proinflammatory mechanisms are related with the pathogenesis of stress-related disorders [48,49]. It has been previously reported that stress induces activation of the microglia and thus an increase of proinflammatory cytokines such as IL-1β, tumor necrosis factor alpha (TNF-α) and interleukin-6 [21,50,51]. Stress-induced cytokines have direct neurotoxic effects on the brain, including brain areas important in the regulation of emotions such as the amygdala and hippocampus [52,53]. The increase of proinflammatory cytokines is also associated with alterations in the signaling pathway of BDNF [54].

In this context, Nurr1 has been proposed as part of the anti-inflammatory pathways in microglia and astrocytes [29]. In our study we did not find differences in *Nurr1* mRNA and *IL-1β* mRNA between the groups, nor at the times analyzed. However, we found a significant increase of Nurr1 and IL-1β protein in the vulnerable group at 24 h after the first exposure to forced swimming. Thus, we suggest that Nurr1 could be acting as a repressor of *IL-1β* expression as previously reported [29] through the physical association of Nurr1 and the p65 subunit of NF-kβ as well as post-translational modifications like phosphorylation and sumoylation [29]. This also supports that Nurr-1 mediated inflammatory signaling is associated with IL-1β regulation [29].

We found no changes in BDNF expression for either mRNA or its protein. This suggests that acute stress, at the first and the second exposure to forced swimming, is not associated with changes in BDNF.

Our results have also shown that the high corticosterone levels at 24 h after first exposure to a forced swim were accompanied by an increase in GR, Nurr1, and IL-1β protein in the vulnerable group as compared to the resilient group which showed opposite results in these parameters. Changes in genomic expression of other molecules of vulnerable and resilient animals in the forced swim have also been reported [35].

Additionally, the immobility behavior in a second exposure to forced swimming persisted showing higher immobility in the vulnerable group vs. resilient. It has been reported that this behavior can persist up to 4 weeks [55] and involves neuroplasticity processes in the hippocampus. In particular, we found no changes in mRNA or protein of GR, Nurr1 and BDNF except an increase in IL-1β in the resilient group after the second exposure to forced swim test.

The non-correlation between the expression of protein and mRNA suggests that changes in protein levels are highly implicated in the stress response, which may involve the participation of post-translational modifications such as phosphorylation and sumoylation. The discordance between mRNA and protein expression has been reported for other molecules in the forced swim test [56]. In addition, it has been shown that during high dynamic phases, such as the stress response, post-transcriptional processes can result in deviations in the relationship between protein and mRNA levels [57].

The current study reports for the first time the relation between GR, Nurr1 and IL-1β with behavioral phenotype in the forced swim test in male rats. Acute stress activates HPA axis leading to glucocorticoid release, as is well known. These exert their action through the GRs (transcription factor) which interact with Nurr1 (transcription factor), that also participates in the stress response, forming the heterodimer Nurr1-GR in hippocampus. In addition, stress-induced neuroinflammation increases both IL-1β and Nurr1. This latter is able to mediate inflammatory responses, signaling the suppression of IL-1β expression. However, our results suggest that both genomic and non-genomic mechanisms, in addition to epigenetic changes and post-translational modifications, could be related to the different behavioral profile. It is important to analyze in future studies the role of these above-mentioned mechanisms in order to identify biomarkers of susceptibility to stress. Likewise, for future research it is necessary to increase the sample size, although our results, according to statistical power, met the requirements to obtain valid conclusions.

Thus, the present results allow us to investigate in future studies the stress response in females. Differences between females (during different stages of the estrous cycle) and males in behavioral response to antidepressant drugs have been reported using the forced swim test [58]. This would help to better understanding the role of GR, Nurr1 and IL-1β in gender. Furthermore, our experiments can be applied to other stress models associated with the intensity and duration of the stressor.

## 4. Materials and Methods

### 4.1. Animals and Bioethical Guidelines

Male Wistar rats purchased from Harlan (Harlan, Mexico) (250–350 g, 3 months of age) were used for the experimental procedures. Rats were housed in groups of 4 per cage, and they were maintained in standard conditions (12:12 h light/dark cycle, lights on at 07:00 h; 21 ± 1 °C; relative humidity 50–60%) with access to food and water *ad libitum*. All experiments were performed in strict accordance with the guidelines established by the National Institutes of Health Guide (USA) for the Care and Use of Laboratory Animals (NIH Publications No. 80-23, revised 1996). In addition, the experimental protocols were approved in accordance with the national regulations (NOM-062-ZOO-1999) specified by the Animal Care and Use Committee of the National Institute of Neurology and Neurosurgery (approval numbers 39/13 and 40/21). Rats were habituated for a week to the housing conditions. In addition, they were handled for a week before the day of the forced swimming test, to reduce extraneous stress factors. All efforts were made to reduce the number of animals used and their suffering. The experiments were performed between 09:00 and 15:00 h in the light phase of the rat’s circadian cycle. The animals were exposed for a habituation time of 1 h to the experimental room before the assays.

### 4.2. Acute Stress Induced by Forced Swimming

Forced swimming is one of the most widely used stress paradigms [31] as well as for evaluating antidepressant-like responses to drugs [59]. Coping with induced-swimming stress elicits the active behaviors of climbing and swimming, and the passive behavior of immobility (floating) [60].

For a comprehensive analysis of the individual behavioral differences induced by acute stress, as well as the subsequent molecular changes, rats were exposed to a single forced swimming session for 15 min and sacrificed after the following recovery times: 0 min (immediately sacrificed), 30 min, 3 h, and 24 h. A control group consisted of unstressed rats, which remained in their home cages until sacrifice. Acute stress was induced as previously reported [59]. Rats were individually placed in an acrylic cylinder (diameter 25 cm and height 50 cm) filled with tap water (water depth of 30 cm, 25 °C ± 1 °C) for 15 min.

We included an additional group of animals with two exposures to forced swimming in the cylinder described above. The first exposure was for 15 min and after 24 h the rat was exposed to a second 5-min swim.

The forced swimming test was videotaped for subsequent behavioral analysis. The procedure was performed between 10:00 h and 12:00 h.

The behavioral response of the rats was analyzed blindly by a trained observer using Kinoscope 3.0.4 software [61]. The time of immobility (indicator of behavioral despair) was evaluated. This behavior was defined as the rat floating passively, keeping its head above the surface, with only one paw showing slight movements. For an integral analysis, climbing and swimming behaviors were also measured. Climbing was defined as the rat making upward-directed movements of the forepaws above the water surface. Swimming was registered as active swimming movements greater than necessary to maintain its head above water.

**Figure 6:**
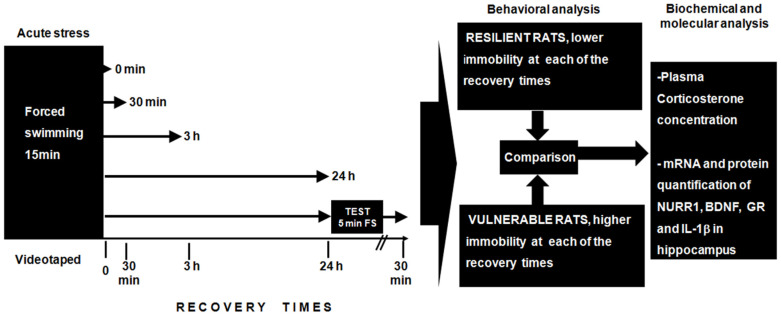
Schematic representation of the experimental procedures performed. BDNF, brain-derived neurotrophic factor; FS, forced swimming; GR, glucocorticoid receptor; Hp, hippocampus; IL-1β, interleukin-1 beta.

It should be noted that the rats were exposed to a single forced swimming session, to classify an individual behavioral phenotype to acute stress. Therefore, according to their behavioral response rats were classified as vulnerable (high immobility) or resilient (low immobility), at each of the recovery times (Figure 6). After collecting the measurements, the animals were classified based on the median immobility time. Rats above this value were designated as vulnerable, while the rest of the rats were assigned to the resilient group. This classification has been previously reported mainly based on the behavioral response during a second forced swim session of 5 min [35,36]. On this basis, we identify two clearly divergent groups in terms of behavioral response.

All rats were rapidly sacrificed by decapitation. A trunk blood sample was immediately collected to quantify plasma corticosterone concentration, and the entire hippocampus was dissected out as previously described [59] to analyze changes in the expression of the genes of interest (Figure 6). The hippocampal tissue was dissected, transferred to liquid nitrogen and subsequently stored at −80 °C to preserve molecular integrity. The RNA and protein total were extracted simultaneously from the complete hippocampus using an AllPrep DNA/RNA/protein Mini Kit (Quiagen, Crawley, UK) following the manufacturer’s instructions.

### 4.3. Quantification of Plasma Corticosterone Concentrations after Acute Stress

The trunk blood samples (5 mL) collected in tubes containing 0.1 M sodium citrate (10:1, *v*/*v*) were centrifuged (2000× *g* for 10 min at 4 °C) to separate the plasma. Aliquots of plasma were stored at −20 °C. Afterward, samples were thawed and diluted 1:20 with sterile water to quantify the plasma corticosterone concentrations. According to the protocol of the enzyme-linked immunosorbent assay (ELISA) kit (corticosterone ELISA kit, Enzo Life Sciences, New York, NY, USA), the quantification was performed in duplicate as previously reported [59]. ELISA plates were read at 405 nm on a microplate spectrophotometer (Epoch, BioTek, Winooski, VT, USA). The intra-assay coefficient of variation was between 6.6% and 8% and the inter-assay coefficient of variation between 7.8% and 13.1%. Plasma corticosterone concentration was determined as percentage bound by using a standard curve ranging from 32 pg/mL to 20,000 pg/mL (kit sensitivity of 27 pg/mL). Values were expressed as nanograms of plasma corticosterone per ml (ng/mL).

### 4.4. mRNA Quantification of Nr3c1, Nurr1, IL-1β, and BDNF

The mRNA expression of *Nr3c1* (gene that encodes for GR), *Nurr1*, *IL-1β*, and *BDNF* in the hippocampus was analyzed and compared between the vulnerable and resilient rats.

The total RNA (1 µg) was reverse transcribed using moloney-murine leukemia virus reverse transcriptase (M-MLV RT; Invitrogen Life Technologies, Carlsbad, CA, USA), according to the manufacturer’s instructions. The obtained cDNA was diluted 1:10 and used in analyzing gene expression by quantitative polymerase chain reaction.

The levels of expression of mRNA hippocampal *Nr3c1, Nurr1*, *IL-1β* and *BDNF* were evaluated in a Rotor-Gene 6000 instrument (Corbett Research Pty Ltd., Sydney, Australia). All values were normalized to *Ywhaz* (gene that encodes for Tyrosine 3-Monooxygenase/Tryptophan 5-Monooxygenase Activation Protein, zeta) expression levels. The house-keeping gene *Ywhaz* is one of the most frequently used and stably expressed reference genes [62,63]. Primer sequences are shown in Table 2.

Each assay was realized with Kapa Sybr Fast Universal quantitative polymerase chain reaction Mix (Kapa Biosystems, Wilmington, MA, USA) and corresponding pair-primer. The total reaction volume was 15 µL and the reactions were performed in triplicate. The relative fold changes were determined by the method of 2^−∆∆Ct^ as described previously [64].

### 4.5. Protein Quantification of GR, Nurr1, IL-1β, and BDNF

The protein expression of GR, Nurr1, IL-1β, and BDNF in the hippocampus was analyzed and compared between the vulnerable rats and resilient rats.

The total protein of the hippocampus was quantified using a bicinchoninic acid test (Sigma-Aldrich, St. Louis, MO, USA). Samples that contained 30 µg of the total were separated by electrophoresis on a 15% polyacrylamide gel (2.5 h at 100 V) and transferred to a nitrocellulose membrane. Nonspecific binding sites were blocked by immersion of the membrane in PBS-Triton 1% containing 5% milk for 2 h at room temperature, and then incubated overnight at 4 °C with primary antibody: anti-Nurr1 (1:1000; N1404 Abcam, Cambridge, MA, USA), anti-BDNF (1:500; EPR1292 Abcam, Cambridge, MA, USA), anti-IL-1β (1:500; ab9722 Abcam, Cambridge, MA, USA) and anti-GR (1:50, SC-568511 Santa Cruz Biotechnology, Dallas, TX, USA). After rinsing three times in PBS-triton 1% for 10 min, the membrane was incubated for 1 h at room temperature with a secondary horseradish peroxidise conjugated antibody (111-035-003 anti-rabbit IgG and 115-035-003 anti-mouse IgG, Jackson ImmunoResearch Laboratories, West Grove, PA, USA). The ECL detection system (GE Healthcare, Piscataway, NJ, USA) was used for chemiluminescent detection according to the manufacturer’s instructions and analyzed using ImageJ 1.49 (NIH) software. Beta-actin (1:500, MAB1501, Millipore, Darmstadt, Germany) was used as an internal control and the results were expressed as ratios to Beta-actin.

### 4.6. Statistical Analysis

For the statistical analysis of the data the software for the social sciences version 20 (SPSS, Chicago, IL, USA) was used. Data on passive (immobility) and active (climbing and swimming) behaviors of all recovery time groups were analyzed with a two-way repeated measures ANOVA test, using the individual behavioral phenotype (vulnerable and resilient) as a factor between subjects and time during the first 15 min of forced swimming (0–5 min, 5–10 min, and 10–15 min) as an intra-subject factor. The Greenhouse-Geisser correction was used for non-spherical data. This was followed by a *post hoc* Bonferroni multiple comparison test.

Two-way ANOVA analysis was conducted to assess the effects of recovery time (0, 30 min, 3 h, 24 h, Test + 30 min) and phenotype (resilient and vulnerable), as well as the interaction of these two factors in the plasma corticosterone levels, expression of GR, Nurr1, IL-1β, and BDNF (mRNA and protein) in response to acute stress due to forced swimming. The recovery time factor included the groups after a single forced swimming test (0, 30 min, 3 h, 24 h) as well as the group with a second exposure to forced swimming 24 h after the first exposure (Test + 30 min). Bonferroni test was used for *post-hoc* comparisons.

The group with a second exposure to forced swimming at 24 h after the first 15 min exposure (Test + 30 min) was analyzed for passive (immobility) and active (climbing and swimming) behaviors in the second exposure (5 min) using t-tests for unpaired data. Results are expressed as means ± SEM. Differences between means were considered statistically significant at *p* < 0.05.

## Figures and Tables

**Table 1:** Main effect and interaction of individual behavioral phenotype and different recovery times.

Expression Measure	Individual Behavioral Phenotype (Vulnerable vs. Resilient)			Recovery Times (0, 30 min, 3 h, 24 h, Test + 30 min)			Individual Behavioral Phenotype × Recovery Times		
	F	*df*	*p*	F	*df*	*p*	F	*df*	*p*
*Nurr1* mRNA	0.491	1.58	0.486	0.904	4.58	0.468	0.370	4.58	0.829
*Nr3c1* mRNA	0.279	1.58	0.600	3.575	4.58	**0.011**	4.397	4.58	**0.004**
*BDNF* mRNA	0.082	1.58	0.776	1.481	4.58	0.220	1.515	4.58	0.210
*IL-1β* mRNA	0.982	1.58	0.326	0.347	4.58	0.845	1.243	4.58	0.303
Nurr1 protein	5.818	1.58	**0.019**	2.343	4.58	0.065	3.042	4.58	**0.024**
GR protein	4.625	1.48	**0.037**	4.889	4.48	**0.002**	0.496	4.48	0.739
BDNF protein	1.404	1.51	0.241	0.630	4.51	0.643	1.539	4.51	0.205
IL-1β protein	0.165	1.55	0.686	2.676	4.55	**0.027**	5.482	4.55	**0.001**

Two-way ANOVA with individual behavioral phenotype and different recovery times as main factor and their interaction. *Nr3c1,* the gene that encodes for GR; BDNF, brain-derived neurotrophic factor; GR, glucocorticoid receptor; IL-1β, interleukin-1 beta; x, interaction; F, F-statistic; df, degrees of freedom; *p*, *p*-value.

**Table 2:** Nucleotide sequences of the primers used in q-PCR.

Genes	Accession Number	Sequence (5′-3′)	Annealing Temp (°C)
*Nurr1*	NM_019328.3	F: AGT CTG ATC AGT GCC CTC GT R: TCA GCA AAG CCA GGA ATC TT	60
*BDNF*	NM_001270631	F: TCC ACC AGG TGA GAA GAG TG R: CGT GGA CGT TTG CTT CTT TC	61
*Nr3c1*	NM_012576	F: CCT CCC ATT CTA ACC ATC CT R: CTC CCT CTG CTA ACC TGT G	60
*IL-1β*	NM_000576.2	F: CAC CTC TCA AGC AGA GCA CAG R: GGG TTC CAT GGT GAA GTC AAC	64
*Ywhaz*	NM_013011.3	F: TTG AGC AGA AGA CGG AAG GT R: GAA GCA TTG GGG ATC AAG AA	60

*BDNF*, brain-derived neurotrophic factor; F, forward; *IL-1β*, interleukin-1 beta; *Nr3c1*, glucocorticoid receptor gene; R, reverse; *Ywhaz*, gene that encodes for Tyrosine 3-Monooxygenase/Tryptophan 5-Monooxygenase Activation Protein, zeta.

## Data Availability

The data supporting the current study are available from the corresponding author upon reasonable request.
